# Association between systemic immune-inflammation index and chronic obstructive pulmonary disease: a population-based study

**DOI:** 10.1186/s12890-023-02583-5

**Published:** 2023-08-10

**Authors:** Chenglin Ye, Li Yuan, Kailang Wu, Bingzheng Shen, Chengliang Zhu

**Affiliations:** 1 Department of Clinical Laboratory, institute of translational medicine, Renmin Hospital of Wuhan University, Wuhan, 430060 Hubei PR China; 2 Department of Clinical Laboratory, Zhongnan Hospital of Wuhan University, Wuhan, 430060 Hubei PR China; 3 State Key Laboratory of Virology, College of Life Sciences, Wuhan University, Wuhan, 430072 Hubei PR China; 4 Department of Pharmacy, Renmin Hospital of Wuhan University, Wuhan, 430060 Hubei PR China

**Keywords:** NHANES, Chronic obstructive pulmonary disease, Cross-sectional study, Population-based study, Systemic immune-inflammation index

## Abstract

**Background:**

The Systemic Immune-Inflammation Index (SII) is a quantitative measurement of the systemic immune-inflammatory response in the human body. The SII has been shown to have prognostic value in various clinical settings, including critical illness, sepsis, and cancer. Its role in chronic obstructive pulmonary disease (COPD) remains unclear and requires further investigation.

**Methods:**

We analyzed demographic data from 16,636 participants in the National Health and Nutrition Examination Survey. Logistic regression analysis was performed to assess the correlation between COPD, lung function, chronic respiratory symptoms and SII. We used Cox proportional hazards (PH) model to analyze the relationship between SII and mortality in COPD patients and healthy individuals. We used propensity score matching (PSM) method to match the COPD population with similar baseline levels with the normal population to further analyze the correlation between SII and COPD.

**Results:**

We recruited 16,636 participants, ages 40 and above, for the study. A multivariable logistic regression analysis revealed that a higher SII level was independently associated with an elevated likelihood of COPD (Odds Ratio (OR) = 1.449; 95% Confidence Interval (CI): 1.252–1.676, *P* < 0.0001) after controlling for all other factors. Results of subgroup analysis showed a significant positive correlation between SII and COPD in different age groups, gender, Body Mass Index, smoking status, and those with a history of hypertension. The SII index had positive correlation with COPD after PSM (OR = 1.673; 95%CI: 1.443–1.938). After full adjustment, an increase in the SII is associated with a higher all-cause mortality rate. The hazard ratio (HR) with a 95% CI in the general population, COPD patients, and healthy individuals are 1.161 (1.088, 1.239), 1.282 (1.060, 1.550), and 1.129 (1.055, 1.207), respectively.

**Conclusions:**

Higher SII levels are linked to higher prevalence of COPD. COPD patients with a higher SII levels have a higher risk of all-cause mortality. Additional large-scale, long-term studies are necessary to confirm these results.

**Supplementary Information:**

The online version contains supplementary material available at 10.1186/s12890-023-02583-5.

## Background

Chronic Obstructive Pulmonary Disease (COPD) is a prevalent respiratory condition characterized by progressive and irreversible airflow limitation [1]. According to the World Health Organization, COPD is estimated to affect over 330 million individuals globally and is projected to become the third leading cause of death by 2030 [2]. Globally, COPD is a major contributor to morbidity and mortality, significantly impacting patients’ quality of life and leading to increased healthcare utilization [3]. Although COPD can affect individuals of all ages, it is most commonly diagnosed in those over the age of 40 [4]. The disease is more prevalent in males, but the incidence in females is increasing, and the gender gap is narrowing. COPD is more prevalent in low- and middle-income countries, where exposure to indoor and outdoor air pollution is high [5]. The pathogenesis of COPD is complex and multifactorial, involving a combination of environmental and genetic factors [6]. Despite significant advancements in our understanding of the disease, further research is needed to better grasp the underlying mechanisms and develop effective interventions that can improve outcomes for COPD patients.

The Systemic Immune-Inflammation Index (SII) is a multi-marker index that provides a comprehensive measurement of the systemic immune-inflammatory response in the human body [7]. SII, based on lymphocyte, neutrophil, and platelet counts, predicts Hepatocellular Carcinoma (HCC) patient recurrence and survival post-surgery independently [7]. Studies show SII objectively reflects inflammation-immunity balance in malignant tumor patients [8, 9] and serves as a prognostic indicator in carcinoma research [10, 11]. Elevated SII levels have been associated with worse prognosis and higher mortality in patients with cancer and cardiovascular disease [12, 13]. Some studies have suggested that SII serves as a marker of chronic inflammation, indicated by increased neutrophil and platelet counts and decreased lymphocyte counts [7, 14]. There is still a lack of large sample studies on the association between SII and COPD.

COPD is distinguished by persistent airway inflammation and immune dysfunction [15]. Elevated levels of pro-inflammatory cytokines and oxidative stress have been observed in the airways of patients with COPD, indicating a persistent state of inflammation [16, 17]. Additionally, COPD is associated with alterations in the immune system, including changes in the balance between T-helper type 1 (Th1) and T-helper type 2 (Th2) cells, as well as changes in the number and function of immune cells such as macrophages and dendritic cells [18–20]. Studies demonstrate a close correlation between immunity and inflammation with the onset and progression of COPD. Given the SII is easily obtainable in clinical settings, exploring its correlation with COPD bears significant importance for the prevention and treatment of COPD. Our study uses the 1999–2010 National Health and Nutrition Examination Survey (NHANES) to investigate the correlation between SII and COPD.

## Methods

### Study data and population

The National Health and Nutrition Examination Survey (NHANES) is a continuous survey of the nutritional status of non-institutionalized Americans. It utilizes nationally representative samples and multi-stage sampling designs to monitor nutritional conditions biennially. The study protocol is approved by the Research Ethics Committee of the National Center for Health Statistics (NCHS). Further information can be found on the NCHS website. All participants in NHANES provided written informed consent. For our study, data from surveys conducted between 1999 and 2010 were analyzed, and demographic information from 35,479 participants was obtained. The lab test results were linked to other NHANES databases using the participant identifier SEQN (the unique sequence number for each participant). The final sample for analysis consisted of 16,636 participants (8,325 males and 8,311 females), after excluding participants with missing data.

### Outcomes

In this study, the diagnosis of COPD relies on the ratio of forced expiratory volume in one second (FEV1) to forced vital capacity (FVC) being less than 0.7 after bronchodilator administration, along with questionnaire surveys of participants and the use of COPD treatment-related drugs such as Long-Acting Muscarinic Antagonist (LAMA) and Long-Acting Beta2-Agonist (LABA). The questionnaire using a composite of three self-reported COPD outcomes (emphysema, chronic bronchitis, and COPD). Participants were considered to have COPD if they answered “yes” to the question, “Have you ever been told that you have emphysema/chronic bronchitis/chronic obstructive pulmonary disease?“ in a standardized medical condition questionnaire, which was administered during a personal interview.

We gathered the mortality data for every participant by connecting with the National Death Index (NDI) until December 31, 2019.

### Calculation and assessment of SII index

SII defined as the product of peripheral platelet count, neutrophil count, and lymphocyte count divided by preoperative lymphocyte count: SII = (P * N) / L, where P, N, L represent peripheral platelet count, neutrophil count, and lymphocyte count respectively.

### Covariates

The study considered various covariates, including demographic factors such as age, gender, and race (classified as non-Hispanic white, non-Hispanic black, Mexican American, or others), educational attainment (categorized as less than high school, high school, or more than high school), and family poverty income ratio (PIR; divided into < 1.85 and ≥ 1.85). Other health variables that were taken into account included smoking status (categorized as never, former, current), BMI (divided into under 25 kg/m^2^, and ≥ 25 kg/m^2^), history of hypertension, diabetes and cardiovascular disease (CVD), white blood cell (WBC) counts and C-reactive protein (CRP).

### Statistical analysis

All statistical analyses were performed using appropriate NHANES sampling weights based on guidelines from the Centers for Disease Control and Prevention (CDC), to account for the complex multistage cluster survey design. Continuous variables were summarized as mean with standard error (SE), while categorical variables were presented as proportions. Given the skewed distribution of the SII index, its description included median and quartile values, and logarithmic transformation was applied in regression analysis. We used the chi-squared test for categorical variables and the Student’s t test for continuous variables to evaluate whether there were differences between COPD patients and normal people within the covariates. Using logistic regression, covariate-adjusted odds ratios (ORs) for the SII to COPD were calculated. In model 1, we adjusted age, gender, and race. Model 2 was adjusted for the covariates in model 1 as well as education levels, PIR, smoke status, BMI, history of diabetes, history of hypertension and history of CVD. Model 3 was adjusted for the covariates in Model 2 as well as WBC and CRP.

Subgroup analyses were performed to investigate whether age, gender, smoking status, BMI, history of hypertension, diabetes and CVD influenced the investigated correlations between SII and COPD outcomes. The *p* values for interactions were tested by the likelihood-ratio test. We used the Cox proportional hazards (PH) model to investigate the association between the SII and all-cause mortality in the general population, COPD patients, and healthy individuals. In the PSM analysis, the nearest method was employed to match COPD patients with normal individuals in a 1:1 ratio. Age, gender, race, PIR, education levels, smoking status, BMI, history of diabetes, hypertension and CVD, WBC, and CRP were adjusted as confounding variables during matching. The statistical analyses were conducted with SPSS 25.0 and R 4.1 software. *P* < 0.05 was considered statistically significant.

## Results

**Fig. 1:**
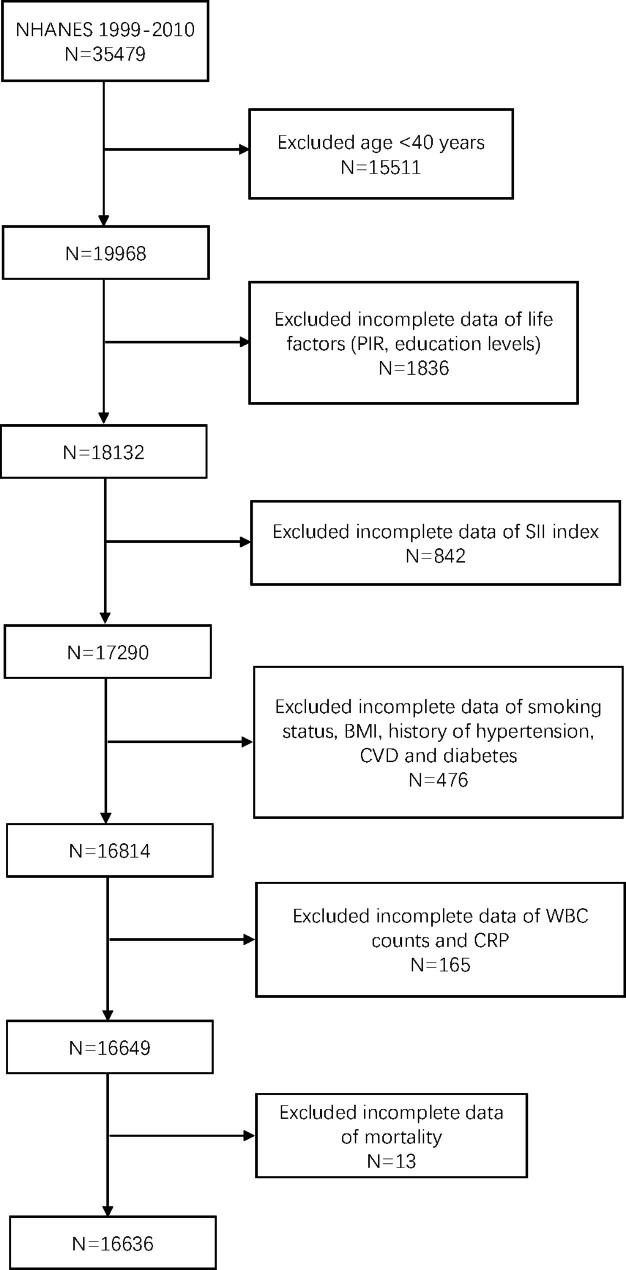
Flowchart of the sample selection from NHANES 1999–2010

The study recruited 16,636 participants, with ages over 40. The sample included 8325 males (50.042%) and 8311 females (49.958%) (Fig. 1). The participants were classified into ethnic groups, with Mexican Americans accounting for 18.328%, non-Hispanic white people 53.889%, non-Hispanic black people 18.532%, and others 9.251%. Table 1 illustrated characteristics of participants. The age of COPD patients was higher than that of normal people, and the difference was statistically significant(*P* < 0.0001). Furthermore, COPD group and normal group had significant differences in these variables: race, education levels, PIR, smoking status, history of diabetes, hypertension and CVD, WBC counts, CRP, and SII index(*P* < 0.01).

**Table 1:** Basic characteristics of participants (n = 16,636) in the NHANES 1999–2010

Outcomes	Total	Normal (N = 15,582)	COPD (N = 1054)	*P*
Age	56.623(0.189)	56.322(0.190)	61.236(0.428)	< 0.0001
Gender				0.079
Male	8325(50.042)	7719(47.360)	606(50.408)	
Female	8311(49.958)	7863(52.640)	448(49.592)	
Race				< 0.0001
Non-Hispanic White	8965(53.889)	8213(76.299)	752(85.775)	
Non-Hispanic Black	3083(18.532)	2922(9.589)	161(7.004)	
Mexican American	3049(18.328)	2994(5.516)	55(1.192)	
Other	1539(9.251)	1453(8.596)	86(6.028)	
Education levels				< 0.0001
less than high school	2749(16.524)	2591(7.432)	158(9.590)	
high school	2598(15.617)	2380(11.642)	218(17.813)	
more than high school	11,289(67.859)	10,611(80.926)	678(72.597)	
PIR				< 0.0001
<1.85	6651(39.98)	6132(25.746)	519(36.887)	
>=1.85	9985(60.02)	9450(74.254)	535(63.113)	
BMI	28.858(0.080)	28.877(0.084)	28.554(0.296)	0.298
Smoking status				< 0.0001
never	7979(47.962)	7820(50.870)	159(16.795)	
former	5457(32.802)	4919(30.487)	538(48.573)	
now	3200(19.235)	2843(18.643)	357(34.632)	
History of diabetes				0.003
no	11,652(70.041)	10,968(76.621)	684(71.949)	
IFG and IGT	1365(8.205)	1273(7.581)	92(8.503)	
DM	3619(21.754)	3341(15.799)	278(19.548)	
History of hypertension				< 0.0001
no	7465(44.873)	7090(52.318)	375(40.464)	
yes	9171(55.127)	8492(47.682)	679(59.536)	
CVD				< 0.0001
no	13,911(83.62)	13,195(87.880)	716(72.769)	
yes	2725(16.38)	2387(12.120)	338(27.231)	
SII	520.000(377.167,722.917)	515.667(375.667,715.333)	591.500(406.381,860.706)	< 0.0001
WBC	7.138(0.027)	7.109(0.028)	7.585(0.092)	< 0.0001
CRP	0.440(0.008)	0.429(0.007)	0.605(0.044)	< 0.001

NHANES, National Health and Nutrition Examination Survey; COPD, Chronic Obstructive Pulmonary Disease; PIR, Poverty Income Ratio; BMI, Body Mass Index; IFG, Impaired Fasting Glucose; IGT, Impaired Glucose Tolerance; DM, Diabetic Mellitus; CVD, Cardiovascular Disease; SII, Systemic Immune-Inflammation Index; WBC: White Blood Cell; CRP, C-reactive protein

Table 2 displays the correlation between SII index and COPD. The results show a positive association between SII index and COPD. The correlation between SII and COPD was significant in both the crude model (OR = 1.673; 95%CI: 1.443–1.938, *P* < 0.0001), model 1 (OR = 1.595; 95%CI: 1.384–1.837, *P* < 0.0001), model 2 (OR = 1.482; 95%CI: 1.286–1.708, *P* < 0.0001), and model 3 (OR = 1.449; 95%CI: 1.252–1.676, *P* < 0.0001). Tertile categorization of SII was performed in sensitivity analysis. Compared to participants in the lowest tertile of SII, those in the highest tertile had a 43.1% increased risk of COPD in model 3(OR = 1.431; 95%CI: 1.161–1.765, *P* = 0.001). The prevalence of COPD was found to increase with an increase in SII (*P* for trend < 0.001).

**Table 2:** Association Between Systemic Immune-Inflammation Index and COPD

	Crude Model		Model 1		Model 2			Model 3				
	OR (95% CI)	*P*	OR (95% CI)	*P*	OR (95% CI)	*P*		OR (95% CI)		*P*		
Continuous	1.673(1.443,1.938)	< 0.0001	1.595(1.384,1.837)	< 0.0001	1.482(1.286,1.708)	< 0.0001		1.449(1.252,1.676)		< 0.0001		
Categories												
Q1	ref		ref		ref			ref				
Q2	1.016(0.820,1.259)	0.882	1.004(0.809,1.246)	0.971	1.009(0.807,1.260)	0.94		1.002(0.800,1.255)		0.983		
Q3	1.641(1.337,2.014)	< 0.0001	1.571(1.278,1.932)	< 0.0001	1.472(1.195,1.813)	< 0.001		1.431(1.161,1.765)		0.001		
*p* for trend		< 0.0001		< 0.0001		< 0.001				< 0.001		

Crude Model: no covariates were adjusted

Model 1: Adjusted covariates for model 1 included gender age, and race

Model 2: Adjusted covariates for model 2 included the covariates for model 1 plus education levels, PIR, smoke status, BMI, history of diabetes, hypertension and CVD.

Model 3: Adjusted covariates for model 3 included the covariates for model 2 plus WBC and CRP.

COPD, Chronic Obstructive Pulmonary Disease; OR: odds ratio. 95%CI: 95% confidence interval; Q1-3 respectively represent the groups divided according to the quantiles of SII.

We selected years with data on lung function and chronic respiratory symptoms, and analyzed the association between the SII and both lung function and chronic respiratory symptoms (Table S1, S2). In the unadjusted (crude) model, an increase in SII was associated with a significant decrease in FEV1 (beta = -87.599, 95% CI: -155.153 to -20.044, *P* = 0.013).This association remained significant after adjusting for potential confounders in model 1 (beta = -92.878, 95% CI: -149.868 to -35.888, *P* = 0.002) and model 2 (beta = -59.813, 95% CI: -112.957 to -6.669, *P* = 0.030).However, in the fully adjusted model 3, the association was attenuated and no longer statistically significant (beta = -10.058, 95% CI: -71.424 to 51.309, *P* = 0.730). As for FVC, in the unadjusted (crude) model, an increase in SII was significantly associated with a reduction in FVC (beta = -80.389, 95% CI: -159.942 to -0.836, *P* = 0.048). This relationship remained significant in the adjusted model 1 (beta = -84.635, 95% CI: -142.460 to -26.810, *P* = 0.006), but was no longer statistically significant in model 2 (beta = -45.724, 95% CI: -100.353 to 8.905, *P* = 0.095) or the fully adjusted model 3 (beta = 19.503, 95% CI: -43.074 to 82.079, *P* = 0.515).

We investigated the relationship between the SII and the presence of chronic respiratory symptoms, including frequent cough, frequent phlegm, and wheezing in the past year (Table S3). In the unadjusted model, an increase in SII was significantly associated with an increased odds of having any chronic respiratory symptom (OR = 1.567, 95% CI: 1.326 to 1.851, *P* < 0.0001). This relationship remained significant in model 1 (OR = 1.584, 95% CI: 1.326 to 1.891, *P* < 0.0001) and model 2 (OR = 1.385, 95% CI: 1.162 to 1.651, *P* = 0.001), but was marginally non-significant in the fully adjusted model 3 (OR = 1.218, 95% CI: 0.999 to 1.485, *P* = 0.051). In Table 3, subgroup analyses on COPD and various confounding factors are presented. The results of subgroup analysis showed that the positive association between SII and COPD was significant in different age groups, for both male and female participants, for BMI, and for those with a history of hypertension. Interestingly, stronger association between SII and COPD was observed in participants who were former or current smokers, with the greatest differences noted in the smoking status subgroup (*P* for interaction = 0.025).

**Table 3:** Subgroup analysis for the association between SII and COPD

character	Q1	Q2	Q3	*P* for trend	*P* for interaction
Age group					0.454
40–59 years	ref	0.897(0.638,1.261)	1.456(1.046,2.025)	0.023	
>=60 years	ref	1.162(0.886,1.524)	1.816(1.430,2.307)	< 0.0001	
Gender					0.918
Male	ref	1.056(0.767,1.453)	1.654(1.248,2.192)	< 0.001	
Female	ref	0.987(0.729,1.336)	1.662(1.284,2.152)	< 0.001	
Smoking status					0.025
never	ref	0.693(0.417,1.154)	0.951(0.542,1.668)	0.891	
former	ref	1.033(0.751,1.422)	2.005(1.557,2.583)	< 0.0001	
now	ref	1.358(0.950,1.941)	1.602(1.070,2.399)	0.023	
BMI group					0.929
<25	ref	1.073(0.730,1.577)	1.643(1.153,2.340)	0.008	
>=25	ref	0.995(0.770,1.285)	1.639(1.302,2.062)	< 0.0001	
History of hypertension					0.759
no	ref	1.102(0.785,1.546)	1.717(1.201,2.455)	0.004	
yes	ref	0.945(0.750,1.190)	1.542(1.223,1.944)	< 0.001	
History of diabetes					0.767
no	ref	0.939(0.710,1.243)	1.547(1.185,2.019)	0.001	
IFG and IGT	ref	1.149(0.528,2.498)	1.857(0.908,3.799)	0.08	
DM	ref	1.338(0.813,2.204)	1.961(1.365,2.817)	< 0.001	
History of CVD					0.552
no	ref	1.086(0.849,1.387)	1.735(1.335,2.254)	< 0.0001	
yes	ref	0.931(0.644,1.345)	1.383(0.969,1.975)	0.062	

SII, Systemic Immune-Inflammation Index; COPD, Chronic Obstructive Pulmonary Disease; BMI, Body Mass Index; IFG, Impaired Fasting Glucose; IGT, Impaired Glucose Tolerance; DM, Diabetic Mellitus; CVD, Cardiovascular Disease; Q1-3 respectively represent the groups divided according to the quantiles of SII

**Fig. 2:**
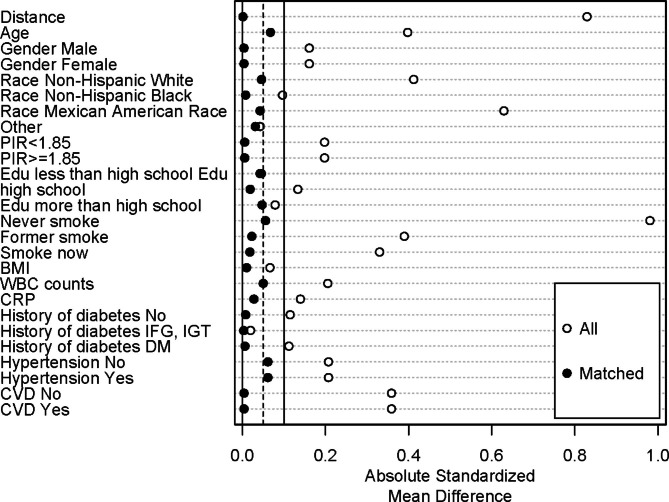
Propensity score matching analysis of the standardized mean difference results for the different variables

**Fig. 3:**
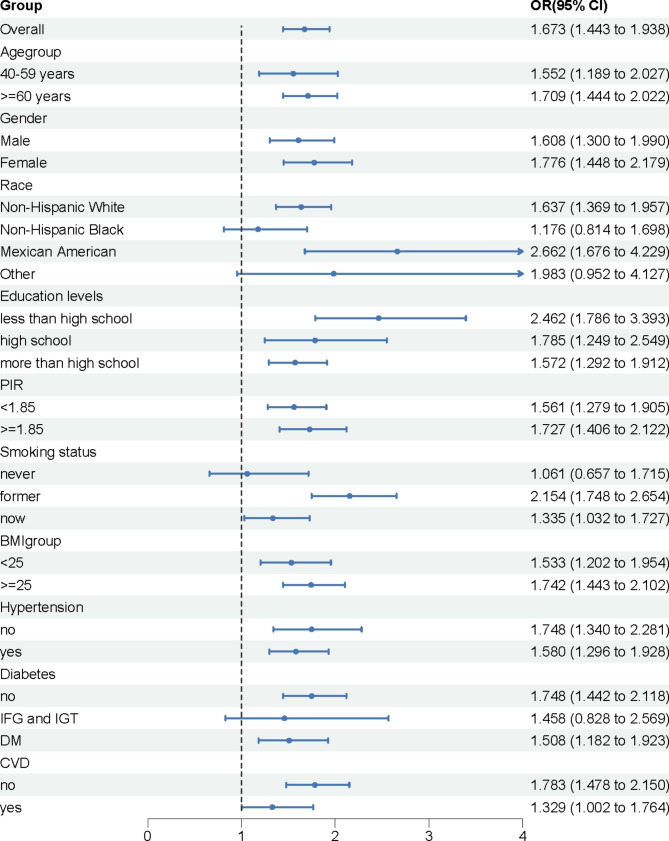
Subgroup analysis for the association between SII and COPD after PSM

At the census day of 31 December 2019, 5193 participants were determined as deceased (31.2%). The median follow-up duration was 146 months (range 0–249 months). We examined the association between the SII and all-cause mortality among three different groups: the general population, patients with chronic obstructive pulmonary disease (COPD), and individuals without COPD (Table 4). The associations were assessed using Cox proportional hazards (PH) models, and the results are presented as hazard ratios (HRs) and 95% confidence intervals (CIs).Among the whole population, after adjusting for potential confounders in model 3, an increase in SII from the first (Q1) to the third quantile (Q3) was associated with a 16.5% increase in the risk of all-cause mortality (HR = 1.165, 95% CI: 1.072–1.267).Among patients with COPD, after full adjustment in model 3, an increase in SII from Q1 to Q3 was associated with a 34.1% increase in the risk of all-cause mortality (HR = 1.341, 95% CI: 1.041–1.727).Among individuals without COPD, after full adjustment in model 3, an increase in SII from Q1 to Q3 was associated with a 13.1% increase in the risk of all-cause mortality (HR = 1.131, 95% CI: 1.036–1.235).These results suggest that an increase in SII is associated with a higher risk of all-cause mortality among both the general population and patients with COPD, highlighting the potential role of SII in predicting mortality. Due to substantial disparities in variables and subject numbers between COPD and normal groups, we conducted a 1:1 PSM analysis (Fig. 2). A total of 2112 participants were enrolled and divided into COPD and normal groups. The baseline characteristics of each group after propensity score matching are presented in Table 5. The baseline characteristics of all participants were comparable after PSM. We found significant differences in SII between COPD and normal groups after PSM and that SII was higher in COPD than in normal group (*P* = 0.002). We then analyzed the association of SII with COPD and its association in different subgroups after PSM (Fig. 3). The SII index had positive correlation with COPD after PSM (OR = 1.673; 95%CI: 1.443–1.938). In the subgroup of patients aged 60 years or more, SII had a stronger association with COPD than in the subgroup of patients aged less than 60 years. SII has a stronger association with COPD in female than in male. Interestingly, we found that SII had a stronger association with COPD among people with lower education levels. SII was also more strongly associated with COPD in the poor population.

**Table 4:** h (95% CIs) for all-cause mortality according to SII among whole population, COPD patients and normal people

Group	Quantiles of SII			Per One-Unit Increment in Natural Log-Transformed SII
	Q1	Q2	Q3	
**Whole population**				
Model 1	ref	0.968(0.893,1.048)	1.272(1.175,1.377)	1.253(1.166,1.347)
Model 2	ref	0.969(0.896,1.048)	1.235(1.139,1.339)	1.223(1.143,1.309)
Model 3	ref	0.956(0.885,1.032)	1.165(1.072,1.267)	1.161(1.088,1.239)
**COPD patients**				
Model 1	ref	1.001(0.740,1.353)	1.455(1.108,1.911)	1.334(1.103,1.615)
Model 2	ref	0.921(0.690,1.230)	1.440(1.116,1.858)	1.390(1.142,1.692)
Model 3	ref	0.912(0.683,1.217)	1.341(1.041,1.727)	1.282(1.060,1.550)
**Normal people**				
Model 1	ref	0.967(0.889,1.053)	1.230(1.132,1.338)	1.216(1.129,1.311)
Model 2	ref	0.977(0.897,1.063)	1.200(1.100,1.311)	1.188(1.106,1.277)
Model 3	ref	0.961(0.884,1.045)	1.131(1.036,1.235)	1.129(1.055,1.207)

Model 1: Adjusted covariates for model 1 included gender age, and race

Model 2: Adjusted covariates for model 2 included the covariates for model 1 plus education levels, PIR, smoke status, BMI, history of diabetes, hypertension and CVD.

Model 3: Adjusted covariates for model 3 included the covariates for model 2 plus WBC and CRP.

HR, Hazard Ratio; 95%CI: 95% confidence interval; SII, Systemic Immune-Inflammation Index; COPD, Chronic Obstructive Pulmonary Disease; Q1-3 respectively represent the groups divided according to the quantiles of SII.

**Table 5:** Basic characteristics of participants after PSM Analysis

Outcomes	Normal (N = 1056)	COPD (N = 1056)	*P*
Age	61.646(0.441)	61.236(0.428)	0.455
Gender			0.932
Male	608(53.177)	606(50.408)	
Female	446(46.823)	448(49.592)	
Race			0.475
Non-Hispanic White	774(85.919)	752(85.775)	
Non-Hispanic Black	158(7.076)	161(7.004)	
Mexican American	45(1.116)	55(1.192)	
Other	77(5.889)	86(6.028)	
Education levels			0.570
less than high school	142(8.248)	158(9.590)	
high school	210(16.928)	218(17.813)	
more than high school	702(74.824)	678(72.597)	
PIR			0.920
<1.85	522(37.614)	519(36.887)	
>=1.85	532(62.386)	535(63.113)	
BMI group			0.288
3<25	305(30.073)	331(31.726)	
>=25	749(69.927)	723(68.274)	
Smoking status			0.501
never	180(18.856)	159(16.795)	
former	526(46.477)	538(48.573)	
now	348(34.667)	357(34.632)	
History of diabetes			0.984
no	688(70.264)	684(71.949)	
IFG and IGT	91(7.078)	92(8.503)	
DM	275(22.657)	278(19.548)	
History of hypertension			0.292
no	344(41.030)	375(40.464)	
yes	710(58.970)	679(59.536)	
CVD			0.936
no	718(73.779)	716(72.769)	
yes	336(26.221)	338(27.231)	
WBC	7.643(0.117)	7.585(0.092)	0.714
CRP	0.600(0.051)	0.605(0.044)	0.932
SII	537.889(386.105,760.769)	591.500(406.381,860.706)	0.002

PSM, propensity score matching; COPD, Chronic Obstructive Pulmonary Disease; PIR, poverty income ratio; BMI, Body Mass Index; IFG, Impaired Fasting Glucose; IGT, Impaired Glucose Tolerance; DM, Diabetic Mellitus; CVD, Cardiovascular Disease; SII, Systemic Immune-Inflammation Index; WBC: White Blood Cell; CRP, C-reactive protein

## Discussion

To the best of our knowledge, this is the first study to have demonstrated a connection between SII and outcomes associated with COPD based on data from a representative national sample. Our findings demonstrate a positive correlation between SII and COPD, even after adjusting for various covariates among the US population.

Extensive research has explored the relationship between immunity and COPD. Studies indicate elevated levels of CD8^+^ T lymphocytes in the blood and airway tissues of COPD patients, alongside increased numbers of activated CD4^+^ and CD8^+^ cells expressing nuclear factor kappa-light-chain-enhancer of activated B cells (NF-κB), signal transducer and activator of transcription 4 (STAT-4), interferon-γ (IFN-γ), and perforin [21, 22]. Stable COPD patients exhibit an increase in sputum CD8^+^ cells compared to control smokers with normal lung function and non-smokers [23]. Studies have shown that in stable COPD patients, the bronchial mucosa is dominated by T lymphocytes, particularly CD8^+^ cells [21, 22, 24]. The reduced apoptosis of CD8^+^ T lymphocytes contributes to their accumulation in the airway submucosa of smokers with COPD [25]. In contrast, studies indicate that the T cell-mediated immune response may be altered or weakened in severe COPD patients [26]. Furthermore, neutrophil accumulation in the sputum of stable COPD patients has been linked to the heightened expression of macrophage inflammatory protein 1α (MIP-1α) in the bronchial epithelium of those with severe disease, as compared to those with mild/moderate COPD [21, 22, 24]. Elevated levels of neutrophils have been observed in the small airways of COPD patients, as the severity of the disease increases, compared to smokers with normal lung function as controls [27]. There was a study indicate that platelet activation may serve as a novel connection between COPD, inflammation, and cardiovascular disease [28]. Platelet activation has been shown to predict adverse outcomes in patients with stable coronary disease and identify individuals at risk for recurrent cardiovascular events following percutaneous coronary intervention [29, 30]. The interplay between platelets and inflammatory cells leads to the release of chemokines, thereby promoting the accumulation of immune mediators, a crucial factor in the formation of atherosclerotic plaques. Studies indicate that platelet activation contributes to structural changes in the pulmonary vasculature, which may be implicated in the pathogenesis of various forms of pulmonary arterial hypertension [31]. These studies have shown that neutrophils, platelets and lymphocytes are closely related to the biological mechanism of COPD.

Several studies have indicated the prognostic value of neutrophil-to-lymphocyte ratio (NLR), platelet-to-lymphocyte ratio (PLR), and the proportion of lymphocytes in COPD patients. Studies indicate that during acute exacerbation of COPD (AECOPD), inflammation severity significantly increases, resulting in higher levels of NLR and PLR. These markers of inflammation might be used to predict the prognosis of COPD patients [32]. Compared with NLR and PLR, SII more comprehensively integrates the relationship between neutrophils, lymphocytes, and platelets. Although SII is a novel marker, studies have explored its relation to lung diseases. Notably, SII holds potential as a prognostic predictor for patients with metastatic non-small-cell lung cancer (NSCLC). Low SII was linked with a longer progression-free survival and overall survival [33]. Another study suggests that a SII value above 500 in patients with connective tissue disorders can indicate pulmonary interstitial involvement [34]. A study found that SII values exceeding 851.51 × 10^9^/L were an independent risk factor for venous thromboembolism (VTE) related to lung cancer [35]. Our study found that SII had positive correlation with COPD after adjusted with various covariates. We used PSM to match the COPD population and the normal population with the same baseline characteristics, and then found that SII was still associated with COPD.

We observed an association between the SII and lung function. In the initial unadjusted model, we found that an increase in SII was significantly associated with a decrease in FEV1, which is a measure of the amount of air forcefully exhaled from the lungs in one second. This suggests that as SII increases, lung expiratory capacity significantly decreases. Even after adjusting for potential confounding factors in models 1 and 2, this association remained statistically significant, indicating that these factors did not substantially alter the relationship between SII and FEV1. However, when we further accounted for all potential confounding factors in the fully adjusted model 3, the association between SII and FEV1 was notably weakened and no longer statistically significant. This implies that other factors may be playing a moderating role in the relationship between SII and lung function. Similar trends were observed in relation to FVC, which measures the maximum amount of air that can be forcibly inhaled after a full exhalation. In the unadjusted model, an increase in SII was significantly associated with a reduction in FVC. The association remained statistically significant in model 1, even after adjusting for potential confounding factors. However, in models 2 and the fully adjusted model 3, the association between SII and FVC lost its statistical significance. This indicates that after considering all potential confounding factors, the relationship between SII and FVC partially disappears. We also investigated the relationship between SII and chronic respiratory symptoms, including frequent cough, frequent phlegm, and wheezing in the past year. In the unadjusted model, an increase in SII was significantly associated with an increased likelihood of experiencing any chronic respiratory symptom. This significant association persisted in models 1 and 2, but in the fully adjusted model 3, the relationship became marginally non-significant. This suggests that part of the association between SII and chronic respiratory symptoms can be explained by certain potential confounding factors. This study reveals a complex relationship between SII and lung function as well as chronic respiratory symptoms. While an unadjusted analysis shows significant associations, accounting for potential confounding factors diminishes or eliminates the statistical significance. These findings indicate that SII alone may not be the sole contributor to changes in lung function and respiratory symptoms, and other biological and environmental factors likely play a role. Further research will be essential to better understand the underlying mechanisms and potential therapeutic implications related to SII, lung function, and respiratory health.

In our study, we found a significant association between the SII and all-cause mortality rates. Our findings suggest a similar predictive role for SII in mortality among both COPD patients and the general population. Increased SII may reflect a heightened state of systemic inflammation and immune activation, which are crucial drivers of disease progression and adverse health outcomes. Chronic inflammation, as indicated by elevated SII, may precipitate tissue damage and organ dysfunction, thus increasing the risk of mortality. However, the exact mechanisms through which SII impacts mortality warrant further investigation. Moreover, prospective studies and randomized trials are needed to validate these associations and explore the therapeutic implications of modulating SII.

Our study has several key strengths. Firstly, the statistical analysis is comprehensive, incorporating a large sample size representative of the national population, precise measurement of SII index, and thorough evaluation of COPD outcomes. Secondly, we considered several influential confounding factors such as demographics and lifestyle habits to eliminate any biases in our results. Finally, we used PSM to match populations with similar baseline characteristics. PSM can increase the efficiency of the statistical analysis by reducing the number of confounders that need to be controlled for in the analysis, compared to traditional regression-based approaches. Our study also has a few limitations. Firstly, the sample population is limited to Americans and cannot be generalized to other populations. Secondly, the age range of participants (over 40 years old) excludes young people and adolescents. Lastly, the cross-sectional design of the study does not allow us to establish a causal link between SII exposure and COPD.

## Conclusions

Our findings showed that higher SII levels are linked to higher prevalence of COPD. COPD patients with a higher SII levels have a higher risk of all-cause mortality. However, additional large-scale, long-term studies are necessary to confirm these results.

### Electronic supplementary material

Below is the link to the electronic supplementary material.

Supplementary Material 1

## Data Availability

The datasets analyzed during the current study are publicly available in the National Health Nutrition Survey (NHANES), https://www.cdc.gov/nchs/nhanes/index.htm.

## Abbreviations

AECOPD: Acute exacerbation of COPD
BMI: Body Mass Index
CDC: Centers for Disease Control and Prevention
CI: Confidence interval
COPD: Chronic obstructive pulmonary disease
CRP: C-reactive protein
CVD: Cardiovascular Disease
DM: Diabetic Mellitus
FEV1: Forced expiratory volume in one second
FVC: Forced vital capacity
HCC: Hepatocellular Carcinoma
HR: Hazard Ratio
IFG: Impaired fasting glucose
IFN-γ: Interferon-γ
LABA: Long-Acting Beta2-Agonist
LAMA: Long-Acting Muscarinic Antagonist
MIP-1α: Macrophage inflammatory protein 1α
NCHS: National Center for Health Statistics
NF-κB: Nuclear factor kappa-light-chain-enhancer of activated B cells
NHANES: National Health and Nutrition Examination Survey
NLR: Neutrophil-to-lymphocyte ratio
NSCLC: Non-small-cell lung cancer
OR: Odds ratio
PIR: Poverty income ratio
PLR: Platelet-to-lymphocyte ratio
PSM: Propensity score matching
SE: Standard error
SII: Systemic Immune-Inflammation Index
STAT-4: Signal transducer and activator of transcription 4
Th1: T-helper type 1
Th2: T-helper type 2
VTE: Venous thromboembolism
WBC: White blood cell
